# Development of Thermodynamic Criteria for Determining the Composition of Duplex Stainless Steels with High Corrosion Resistance

**DOI:** 10.3390/ma17020294

**Published:** 2024-01-07

**Authors:** Aleksandr Fedorov, Vladimir Karasev, Pavel Kovalev, Nikita Shaposhnikov, Andrey Zhitenev

**Affiliations:** 1 Scientific and Technical Complex «New Technologies and Materials», Peter the Great St. Petersburg Polytechnic University, 195251 St. Petersburg, Russia; fedorov_as@spbstu.ru (A.F.); karasev_vs@spbstu.ru (V.K.); shaposhn_no@spbstu.ru (N.S.); 2 Institute of Machinery, Materials, and Transport, Peter the Great St. Petersburg Polytechnic University, 195251 St. Petersburg, Russia; kovalev_pv@spbstu.ru

**Keywords:** duplex stainless steels, pitting resistance equivalent number, thermodynamic criteria, crevice corrosion, chromium nitrides, niobium carbonitrides, corrosion properties

## Abstract

One of the most popular methods for ranking duplex stainless steels (DSSs) and predicting their corrosion properties is the calculation of the pitting resistance equivalent number (PREN). However, since DSSs are two-phase materials with a significant fraction of secondary phases and precipitates, the application of the PREN can be highly limited. This article attempted to use a new approach to describe the corrosion resistance of these steels. The corrosion resistance of two DSSs of the same class was investigated. Under identical solution heat treatments in the temperature range of 1050–1200 °C, the crevice corrosion resistance of one steel increased, while that of the other decreased. It was demonstrated that the amounts of austenite and ferrite changed similarly in these steels, and the different corrosion resistances were associated with the behaviors of secondary phases: niobium carbonitride and chromium nitride. SEM-EDS analysis was conducted to analyze the redistribution of elements between phases in both cases, showing good agreement with the thermodynamic modeling results. The PREN was calculated for each phase depending on the treatment temperature, and a method for calculating the effective PREN (*PREN_eff_*), accounting for phase balance and secondary phases, was proposed. It was shown that this indicator described corrosion properties better than the classical PREN calculated for the average steel composition. This study demonstrated how the calculation of critical temperatures (the temperature of equal amounts of ferrite and austenite, the temperature of the beginning of chromium nitride formation, and the temperature of the beginning of σ-phase formation) could describe the corrosion resistance of DSSs. Maximum possible deviations from these temperatures were defined, allowing the attainment of the required corrosion properties for the steels. Based on the conducted research, an approach for selecting new compositions of DSSs was proposed.

## 1. Introduction

One of the most common methods for predicting the corrosion properties of duplex stainless steels (DSSs) is the calculation of the pitting resistance equivalent number (PREN). The PREN was initially developed for austenitic stainless steels [1]. For this class of materials, the use of regression equations describing properties based on chemical composition is justified, as it is a single-phase material where practically all elements are in a solid solution. The initial equation of the PREN considered only chromium and molybdenum; however, with the development of alloy compositions for austenitic steels, the influences of manganese [2,3], niobium [4], tungsten [5], carbon [6], and other elements were considered. One of the most common equations, widely used by many authors, is provided in [6], where the positive contributions of chromium, molybdenum, tungsten, copper, and nitrogen are considered, while carbon has a negative contribution:

PREN = %Cr − 14.5 × %C + 3.3 × %Mo + 2 × %W + 2 × %Cu + 16 × %N(1)

Unlike austenitic stainless steels, duplex stainless steels contain both austenite and ferrite, which significantly differ from each other in chemical composition and, consequently, possess their individual PRENs [7,8]. Moreover, depending on the temperature of the heat treatment, the ratio of the volume fractions of austenite and ferrite, their chemical compositions, and, consequently, the PREN of each phase change. It is also crucial to mention that in corrosive environments, austenite and ferrite form a microgalvanic couple [9], and depending on the environment’s composition, both austenite and ferrite can act as an anode or cathode, determining the corrosion resistance of the entire material. Another significant distinction of DSSs from austenitic stainless steels is that these alloys, due to a high level of nitrogen and nitride-forming elements, tend to form various secondary phases. These phases actively participate in the redistribution of chemical elements, creating new interphase boundaries and thereby becoming active contributors to corrosion processes [10,11].

However, even in accepted standards, such as ISO 17781 [12], the classification of DSSs based on the PREN indicator is adopted (Table 1). This classification is quite formal, and, for example, the study in [13] expresses the opinion that an increase in the PREN level indicates only an increase in the steel’s alloying level and does not reflect an objective change in corrosion properties.

Therefore, to describe and predict the corrosion properties of specific grades of DSSs, it is advisable to explore other approaches. In earlier studies by the authors, an attempt was made to develop a methodology for selecting the chemical composition and processing technology, considering the prediction of the phase composition via thermodynamic modeling [14]. Critical points on the phase diagram were proposed, the positions of which relative to the temperatures of a particular treatment determine the final properties of the product:

- $T_{50/50}^{\delta /\gamma }$—the temperature at which austenite (γ) and ferrite (δ) are present in equal proportions. It is essential to note that 50/50 primarily signifies an *equal* amount of austenite and ferrite, which does not necessarily have to be 50 vol.% each.
- $T_{{Cr}_{2}N}^{0}$ and $T_{\sigma }^{0}$—the temperatures at the beginning of the formation of chromium nitride and the σ-phase, respectively.

In addition, the criteria for the performance of DSSs were formulated: $T_{50/50}^{\delta /\gamma }$ should not exceed the maximum temperature of the final treatment (hot rolling or heat treatment), and $T_{{Cr}_{2}N}^{0}$ and $T_{\sigma }^{0}$ should be lower than $T_{50/50}^{\delta /\gamma }$ while achieving the maximum possible PREN for the entire alloy.

It is quite evident that under these conditions, the steel should most fully realize the “potential” of corrosion resistance inherent in it at the level of alloying. However, in the conditions of real processes, phase transformations, especially in solid metals, proceed with a certain degree of incompleteness. Therefore, the goal of this study was to determine the temperature intervals relative to the critical points of phase transformations where the performance criteria of DSSs are met and where the PREN can be used for an objective assessment of corrosion properties.

## 2. Materials and Methods

In this study, two cast DSSs produced under industrial conditions in open-induction furnaces were investigated (Table 2). Despite significant differences in their chemical compositions, their PREN values, calculated using Equation (1), are quite close. Both steels can be classified as super DSSs; thus, a similar level of corrosion properties can be expected.

Due to the significant structural and chemical heterogeneity formed during the solidification of DSSs castings and the cooling rate determined with the casting technology, leading to incomplete phase transformations [15], solution heat treatment is performed for castings. To minimize these heterogeneities and achieve varying ratios of austenite and ferrite, the samples were heated in a WiseTherm muffle furnace (Daihan Scientific Co., Ltd., Wonju, Korea) at the temperature range of 1050–1250 °C with a 50 °C increment, holding at each temperature for 60 min, followed by rapid cooling in water (i.e., solution heat treatment). Employing the method described in [16,17] based on selective etching with the Beraha reagent and subsequent automatic analysis using a Thixomet Pro image analyzer (Thixomet Ltd., St. Petersburg, Russia), the contents of ferrite and austenite were assessed. Metallographic studies were performed using optical microscopy methods with a Reichert-Jung MeF3 A microscope (Reichert Inc., Depew, NY, USA).

The corrosion properties of the experimental samples were determined using the gravimetric method after testing for resistance to crevice corrosion according to ASTM G48-11, method B [18]. The specimens were placed inside a beaker and immersed in an FeCl_3_ solution for 72 h at 50 °C. The corrosion rate (*CR*) was calculated using the following Equation (2):

*CR* (g/m^2^·h) = ∆*m*/(*S* · *τ*), (2)

where ∆*m* is the weight loss (g); *S* is the surface area of the exposed sample (m^2^); and *τ* is the immersion time (h).

The local chemical composition was determined using a Tescan Mira scanning electron microscope (Tescan Group, a.s., Brno, Czech Republic) equipped with an energy-dispersive X-ray analyzer (SEM-EDS). Thermodynamic modeling of phase-formation processes was carried out using the Thermo-Calc software (TCW5, Thermo-Calc Software Inc., Solna, Sweden) equipped with the TCFE6 [19] database, as it is the most suitable among the known databases for predicting the phase composition of DSSs [17].

## 3. Results

### 3.1. Corrosion Properties and Microstructures

**Figure 1:**
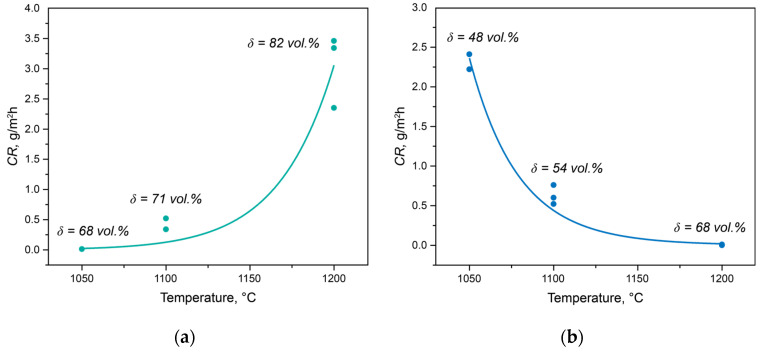
The crevice corrosion rates of Steel 1 (**a**) and Steel 2 (**b**) as a function of solution heat treatment temperature.

The dependencies of the crevice corrosion rates for the experimental steels subjected to solution heat treatment at different temperatures are shown in Figure 1. For Steel 1, as the solution heat treatment temperature increased from 1050 to 1200 °C, the corrosion rate significantly rose from 0.01 to 3.40 g/m²·h (Figure 1a). In the case of Steel 2, the corrosion resistance behavior differed. With the increase in the solution heat treatment temperature, the corrosion resistance, as assessed by the crevice corrosion rate, markedly decreased from 2.32 to 0.00 g/m²·h (Figure 1b).

**Figure 2:**
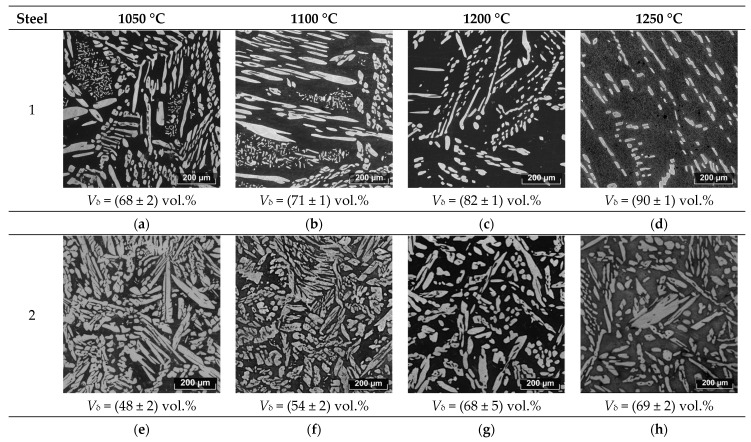
The evolution of the microstructures in the investigated steels depending on the solution heat treatment temperature: (**a**–**d**) Steel 1 at 1050–1250 °C; (**e**–**h**) Steel 2 at 1050–1250 °C.

These results lead to the conclusion that these two steels with similar PREN values and identical solution heat treatments result in significantly different crevice corrosion resistances. The microstructures of the samples after testing were investigated for the interpretation of this phenomenon (Figure 2). Additionally, the microstructural analysis included additional samples after solution heat treatment at intermediate temperatures of 1150 and 1250 °C.

After solution annealing at 1050 °C for 60 min in Steel 1, the fraction of δ-ferrite was 68 vol.%. Austenite was represented by individual elongated grains and colonies of small islands. Increasing the solution annealing temperature to 1100 °C raised the δ-ferrite fraction to 71 vol.%, and the morphology of austenite remained unchanged. Further increasing the solution annealing temperature to 1200 °C led to an increase in the δ-ferrite fraction to 82 vol.%. After this treatment, colonies of small austenitic grains completely disappeared, and the remaining grains decreased in size, losing their sharp angularity due to simultaneous processes of dissolution and Ostwald ripening [20]. With an increase in the solution annealing temperature to 1250 °C, the δ-ferrite fraction increased to 90 vol.%, and the austenite islands further reduced in size, approaching a more rounded shape. Similar trends in structural changes were observed during the solution annealing of Steel 2—the amount of δ-ferrite increased from 48 to 69 vol.%, with the temperature increasing from 1050 to 1200 °C.

Thus, the change in phase balance does not fully explain the change in corrosion properties. To illustrate this, the volume fractions of ferrite for each temperature are plotted in Figure 1. The behavior of Steel 1 is entirely understandable since in a chloride-ion-containing environment during testing, ferrite acts as the anode and actively dissolves [21]. Therefore, with an increase in the volume fraction of ferrite, the anode’s surface area increases, leading to an elevated corrosion rate. The behavior of Steel 2 is entirely unpredictable since its inferior corrosion properties correspond to the best phase balance.

**Figure 3:**
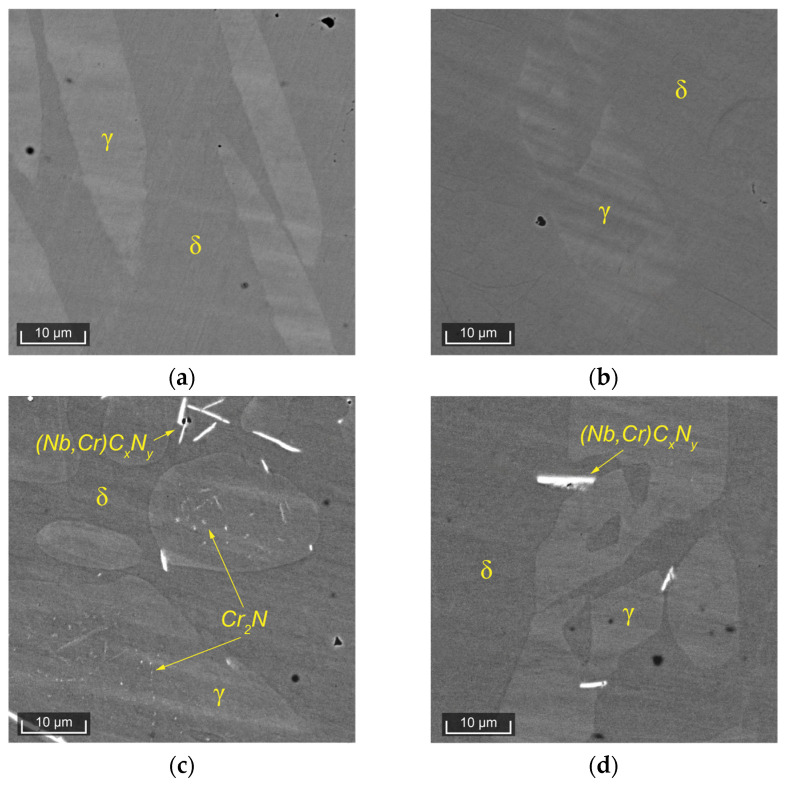
SEM images of (**a**) Steel 1, solution heat treatment at 1050 °C; (**b**) Steel 1, solution heat treatment at 1250 °C; (**c**) Steel 2, solution heat treatment at 1050 °C (fine nitride precipitates in austenite); and (**d**) Steel 2, solution heat treatment at 1250 °C.

The behavior of the corrosion resistance of the two DSSs, seemingly similar in structural changes, is not only due to the change in the quantity of austenite and ferrite but also to other phases that are much more difficult to detect. Figure 3 presents microstructural images of the experimental steels obtained using a scanning electron microscope.

In Steel 1, after the solution heat treatment at 1050 °C, a distinct ferritic matrix and islands of austenite are clearly visible (Figure 3a). After the solution heat treatment at 1250 °C, the microstructure remains the same. In Steel 2, after the solution heat treatment at 1050 °C, in addition to ferrite and austenite, inclusions of niobium carbonitrides and dispersed precipitates, morphologically identifiable as chromium nitrides [22], are well observed (Figure 3c). After the solution heat treatment at 1250 °C, in Steel 2, the quantity of niobium carbonitrides slightly decreases, and dispersed chromium nitrides are hardly distinguishable. These inclusions significantly reduce the corrosion resistance of the alloy. Thus, in the considered DSSs with similar PRENs and nearly identical behavior of ferrite and austenite during heat treatment, the behavior of secondary phases differs, leading to radical differences in corrosion resistance.

### 3.2. Thermodynamic Modeling and Redistribution of Chemical Elements

**Figure 4:**
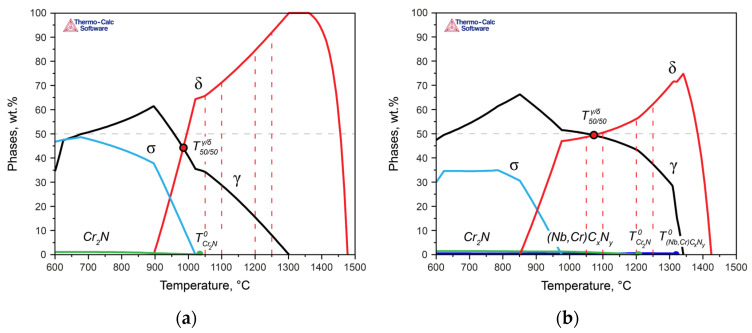
Thermodynamic modeling results for Steel 1 (**a**) and Steel 2 (**b**).

To describe the behavior of phases in the DSSs, thermodynamic modeling of the phase formation processes was carried out (Figure 4). The modeling considered the liquid phase and utilized the associated solution model as well as the sublattice–regular solution model to describe the austenite and carbonitrides (with the parameters of the face-centered cubic lattice), ferrite (with the parameters of the body-centered cubic lattice), and existing chromium nitrides (with the parameters of the hexagonal close-packed lattice) to characterize the behavior of chromium nitride. In this context, carbon was taken into account, understanding that this compound is a complex carbonitride. However, for simplicity, both here and in subsequent discussions, we will refer to it as chromium nitride (Cr_2_N).

According to calculations performed for Steel 1 (Figure 4a), crystallization began with the formation of δ-ferrite dendrites, and after solidification, a polymorphic transformation to austenite occurred in the solid state. In Steel 2, crystallization similarly proceeded with the formation of primary δ-ferrite dendrites, but at the end of crystallization, some amount of austenite was formed via the peritectic–eutectic reaction (Figure 4b) [23]. Upon further cooling, the fraction of austenite increased due to the diffusion growth of γ-crystals and polymorphic transformation of δ → γ. Thus, the results of the thermodynamic modeling fully describe the observed evolution of the structures of the experimental steels during the heat treatment. Additionally, the difference in the crystallization mechanisms, namely, L → δ in Steel 1 and L → δ → δ + γ in Steel 2, explains the different initial morphology of austenite maintained at low heat treatment temperatures (Figure 2a,e).

With a further decrease in temperature, in Steel 1, at 1040 °C, the formation of the chromium nitride became possible, and at 1010 °C, the σ-phase was formed. In Steel 2, which contains significantly more nitrogen than Steel 1, along with niobium, niobium carbonitrides formed at the end of the solidification temperatures, and at 1205 °C, chromium nitrides formed. The σ-phase was formed at 975 °C. 

**Figure 5:**
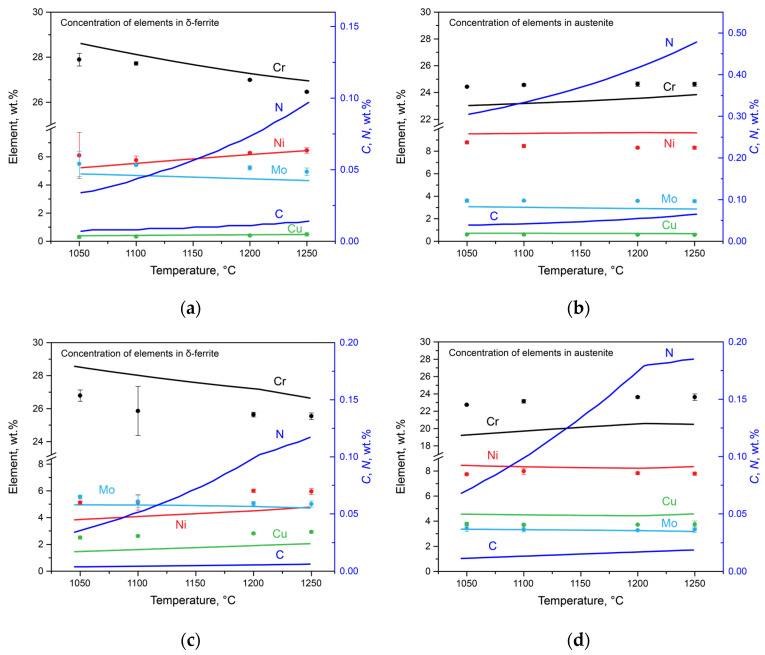
Concentrations of elements in phases as a function of solution heat treatment obtained using SEM-EDS (dots) and Thermo-Calc (lines): (**a**) Steel 1 in ferrite; (**b**) Steel 1 in austenite; (**c**) Steel 2 in ferrite; and (**d**) Steel 2 in austenite.

Simultaneous with the change in the phase composition, there was a redistribution of the alloying elements among austenite, ferrite, carbonitrides, and nitrides. This phenomenon was investigated using thermodynamic modeling and direct measurements using the SEM-EDS method. The results are presented in Figure 5, where the calculated results are represented with lines, and the experimental data are marked by dots. Using SEM-EDS, the chromium, nickel, molybdenum, and copper contents were estimated, and these results are discussed below along with the calculated nitrogen and carbon contents. The data on the concentrations of these elements can only be obtained via calculations.

In Steel 1, with the increase in the temperature of the solution heat treatment from 1050 to 1250 °C, the following changes occurred: in δ-ferrite (Figure 5a), the chromium and molybdenum contents decreased from 27.89 to 26.46 wt.% and from 5.66 to 4.94 wt.%, respectively; the nickel content increased from 6.10 to 6.45 wt.%; and the copper content increased from 0.30 to 0.49 wt.%. In austenite (Figure 5b), the chromium content slightly increased from 24.43 to 24.62 wt.%, and the molybdenum content decreased from 3.60 to 3.56 wt.%. The nickel content decreased from 8.78 to 8.30 wt.%, and the copper content remained almost unchanged at 0.59 wt.%. According to the calculation, the nitrogen content in ferrite increased from 0.03 to 0.10 wt.%, and in austenite, it increased from 0.31 to 0.48 wt.%. The carbon content increased from 0.007 to 0.014 wt.% in ferrite and from 0.039 to 0.065 wt.% in austenite.

For Steel 2, in δ-ferrite (Figure 5c), the concentrations of chromium and molybdenum decreased from 26.53 to 25.44 wt.% and from 5.54 to 5.01 wt.%, respectively, while the amounts of nickel and copper increased from 5.12 to 5.95 wt.% and from 2.50 to 2.92 wt.%, respectively. In austenite (Figure 5d), the chromium concentration increased from 22.73 to 23.62 wt.%, and the molybdenum concentration decreased from 3.42 to 3.33 wt.%. The nickel concentration remained practically unchanged at 7.77%, while the copper concentration increased from 3.77 to 3.83 wt.%. The calculated nitrogen content increased from 0.03 to 0.12 wt.% in ferrite and from 0.07 to 0.18 wt.% in austenite. The carbon content increased from 0.004 to 0.006 wt.% in ferrite and from 0.011 to 0.019 wt.% in austenite.

Despite some differences between the experimental and calculated data, which arose due to the incomplete diffusion processes during transformations [24], the similarity between the results is good. The largest mistake was observed for chromium, which was associated with its lower diffusion coefficient [25].

**Figure 6:**
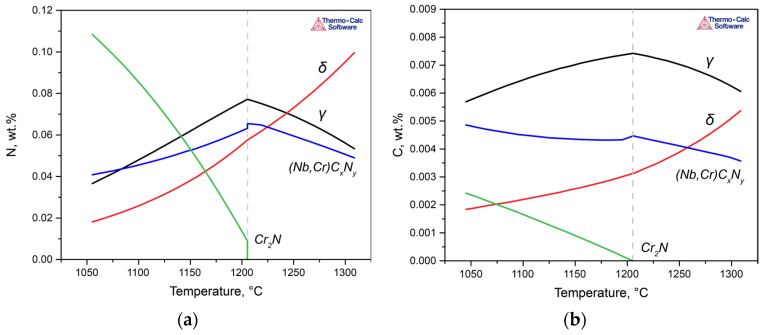
Distributions of nitrogen (**a**) and carbon (**b**) among phases during cooling in Steel 2.

In Steel 2, in addition to ferrite and austenite, the formation of niobium carbonitrides and chromium nitrides occurs at processing temperatures, contributing to the redistribution of nitrogen. Due to these particles, a significant depletion of the solid solution in nitrogen occurs (Figure 6a). The same applies to carbon, which is part of the complex carbonitrides (Figure 6b). However, the situation with carbon is more contradictory. While there is a depletion of ferrite and austenite in carbon, formally increasing the PREN of the phases, the formed particles will degrade the corrosion resistance of the entire alloy due to the emergence of new interphase boundaries. Nevertheless, since these effects are difficult to formally account for, at this stage, it was considered that the contribution of particles to the reduction in corrosion properties was primarily due to the depletion of the solution.

Using the data on the chemical compositions of austenite and ferrite in each steel at each temperature, it is possible to estimate the PREN for each phase, taking into account the redistribution of elements, including between secondary phases. The amounts of nitrogen and carbon were considered, utilizing the values of the limiting solubilities for each phase at the corresponding temperature, estimated using Thermo-Calc calculations (Figure 5). Additionally, to assess the integral characteristics of the entire alloy, this study proposes calculating the effective PREN value for the alloy, considering the volume fractions of austenite and ferrite (3):

*PREN*_*eff*_ = *PREN*_*δ*_ ∙ *V*_*δ*_ + *PREN*_*γ*_ ∙ *V*_*γ*_, (3)

where *PREN*_*δ*_ is the PREN in δ-ferrite; *V*_*δ*_ is the volume fraction of δ-ferrite; *PREN*_*γ*_ is the PREN in austenite; and *V*_*γ*_ is the volume fraction of austenite.

**Figure 7:**
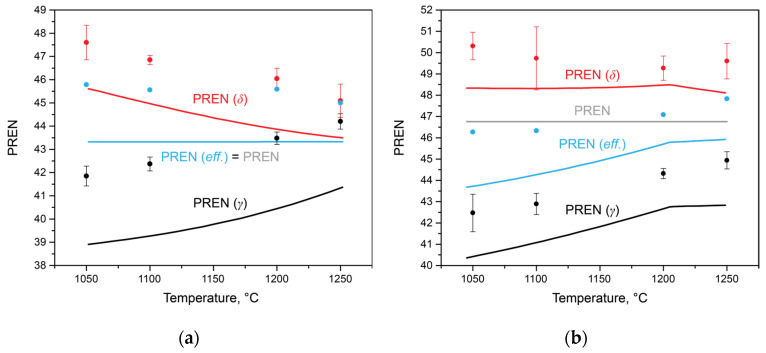
Variation in *PREN*_*eff*_ depending on solution heat treatment, obtained using SEM-EDS (dots) and Thermo-Calc (lines): (**a**) for Steel 1; (**b**) for Steel 2.

This calculation allows for the consideration of phase balance, as well as the formation of secondary phases. The results of these calculations are presented in Figure 7. In Steel 1 (Figure 7a), as the solution heat treatment temperature increased and the amount of δ-ferrite increased from 68 to 90 wt.% (1050 and 1250 °C, respectively), the PREN values calculated for austenite and ferrite changed monotonically. The *PREN*_*δ*_ decreased, primarily due to a decrease in the chromium concentration, while the *PREN*_*γ*_ increased due to an increase in chromium and nitrogen. Since no secondary phases are present in this steel within the temperature range of the considered heat treatments, the *PREN*_*eff*_ remains constant and coincides with the PREN calculated according to Equation (1) for the average composition of the steel. Considering the results of the corrosion tests, it can be concluded that in this steel, the processes of the anodic dissolution of ferrite in a chloride-containing environment play a key role. Therefore, the *PREN*_*δ*_ more effectively describes the overall steel properties than the PREN.

In Steel 2, there is a bend in the curves of changes in the PREN of austenite and ferrite at a temperature of 1205 °C, above which the formation of chromium nitrides is impossible. The *PREN*_*δ*_ value remained constant in the temperature range from 1050 to 1205 °C and then decreased. At the same time, the value of *PREN*_*eff*_, calculated using thermo-calc, also increased from 43.79 to 46.08. However, in all heat treatment intervals, it did not reach the PREN value calculated for the average composition of the steel due to the redistribution of nitrogen and carbon between austenite, ferrite, chromium nitride, and niobium-based carbonitride. Analyzing the data in Figure 7b in conjunction with the corrosion rate values in Figure 1b, it can be concluded that in this steel, with similar PREN values, the processes of precipitate formation play a crucial role in determining corrosion properties. The PREN values calculated for the experimental data are also plotted on the same figure. Considering the measurement error and taking into account the incompleteness of diffusion processes in elemental redistribution, it can be concluded that the trends in their changes correspond well with the calculated data.

### 3.3. Determining Thermodynamic Criteria

**Figure 8:**
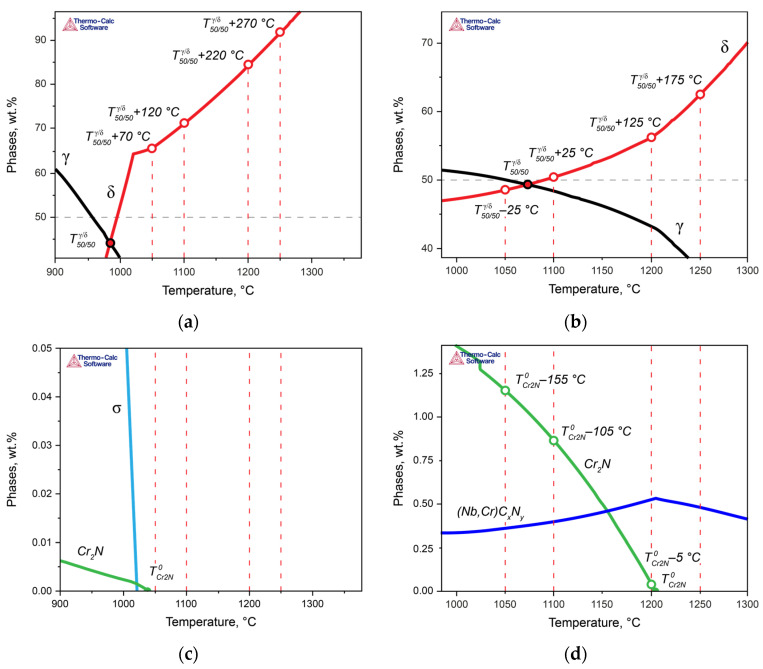
Positions of the developed criteria on thermodynamic curves and their deviations at different temperatures for Steel 1 (**a**,**c**) and Steel 2 (**b**,**d**).

**Figure 9:**
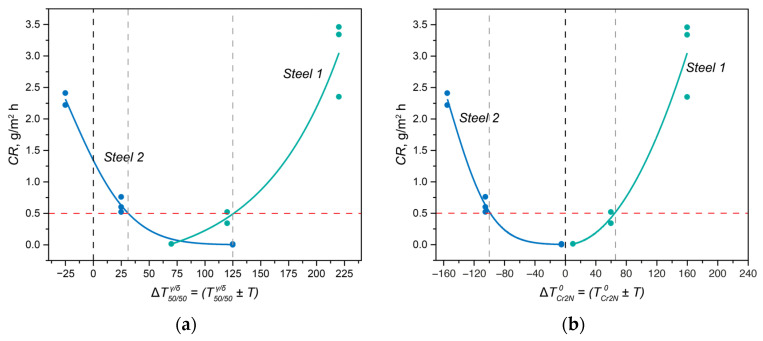
Practical application of the developed criteria $T_{50/50}^{\delta /\gamma }$ (**a**) and $T_{{Cr}_{2}N}^{0}$ (**b**) to describe the change in corrosion properties with variations in the phase composition.

The above analysis demonstrated that for steels with the same PREN level, formally belonging to the same class of corrosion resistance, this indicator does not predict their behavior when assessing their corrosion resistance. Therefore, let us now consider the properties of the experimental steels while taking into account the critical temperatures of the steels: $T_{50/50}^{\delta /\gamma }$, $T_{{Cr}_{2}N}^{0}$, and $T_{\sigma }^{0}$. Since all solution heat treatments were conducted at temperatures above $T_{\sigma }^{0}$, we will not consider this critical point here. For convenience, Figure 8 presents more detailed fragments of Figure 3, in which the critical temperatures of the two steels are marked, and the heat treatment temperatures are indicated as the difference between the actual solution heat treatment temperature and the temperatures $T_{50/50}^{\delta /\gamma }$ (Figure 8a,b) and $T_{{Cr}_{2}N}^{0}$ (Figure 8c,d), respectively. In Figure 9, the dependence of the crevice corrosion rate for the two studied steels is shown, depending on the deviations in the heat treatment temperatures from the critical thermodynamic points.

In Steel 1, at 1050 °C, the solution heat treatment temperature was higher than $T_{50/50}^{\delta /\gamma }$ by Δ*T* = 70 °C (Figure 8a,c and green curves in Figure 9). As the solution heat treatment temperature increased, it moved further away from $T_{50/50}^{\delta /\gamma }$ with an even larger Δ*T*: $T_{50/50}^{\delta /\gamma }$ + 120 °C at 1100 °C, $T_{50/50}^{\delta /\gamma }$ + 220 °C at 1200 °C, and $T_{50/50}^{\delta /\gamma }$ + 270 °C at 1250 °C. Moreover, all solution heat treatment temperatures were above $T_{{Cr}_{2}N}^{0}$, so for all treatment temperatures, Δ*T* was greater than zero, indicating the phase diagram region where chromium nitride was dissolved. Consequently, to ensure high corrosion resistance for this steel, it is essential to primarily maintain a small deviation in the solution heat treatment temperature from $T_{50/50}^{\delta /\gamma }$.

In Steel 2, the situation was different: as the distance from $T_{50/50}^{\delta /\gamma }$ ($T_{50/50}^{\delta /\gamma }$ + Δ*T*) increased, the corrosion rate of the steel decreased (Figure 8b,d and blue curves in Figure 9a). However, due to the high temperature of $T_{{Cr}_{2}N}^{0}$ during annealing at 1050 and 1100 °C, the difference between the actual solution heat treatment temperature and $T_{{Cr}_{2}N}^{0}$ was less than zero, meaning chromium nitride formed and depleted the solid solution of nitrogen. However, when the annealing temperature reached 1200 °C, the ΔT with respect to $T_{{Cr}_{2}N}^{0}$ became approximately –5 °C, practically approaching the region where chromium nitride should dissolve. As a result, the corrosion resistance increased.

The conclusions drawn from these considerations have direct practical applications. When formulating requirements for the corrosion resistance of the developed DSSs, it is necessary to take into account the critical points of the phase diagrams and select chemical compositions in such a way that the actual heat treatment temperatures do not deviate from them by a critical Δ*T*. For example, if the required crevice corrosion rate of the steel should not exceed 0.5 g/m^2^·h for centrifugal pumps used in the petroleum and petrochemical industries, the composition should be chosen with consideration of the *PREN*_*eff*_ so that the following inequalities (4)–(6) are simultaneously satisfied (data from Figure 9):

(4) $$\left|T_{\mathrm{sol.anneal.}}-T_{50/50}^{\delta /\gamma }\right|\;<\;125\;{}^{\circ}C;$$

(5) $$T_{\mathrm{sol.anneal.}}-T_{{Cr}_{2}N}^{0}\;>\;-100\;{}^{\circ}C;$$

(6) $$T_{\sigma }^{0}\;<\;T_{\mathrm{sol.anneal.}}$$

In the given inequalities, the difference between the solution annealing temperature and $T_{50/50}^{\delta /\gamma }$ should be calculated as an absolute value since there may be some fluctuation in the phase composition, toward an increase in either the ferrite fraction or the austenite fraction. The difference between the solution heat treatment temperature and $T_{{Cr}_{2}N}^{0}$ is strictly defined, as it represents the maximum possible undercooling below the temperature of chromium nitride formation, at which intensive nitride formation still does not occur within the actual annealing times. In the case of strongly negative values of ΔT concerning the temperature of $T_{{Cr}_{2}N}^{0}$, it is necessary to maximize not the *PREN*_*eff*_ but the PREN of the phase acting as the anode in the specific test environment (specifically, for chloride-containing solutions, it is the *PREN*_*δ*_). Additionally, it is crucial to consider inequality (6) to exclude the formation of the σ-phase.

The proposed approach can be further extended to other grades of DSSs. Via such analysis, it is possible to more accurately assess the values of Δ*T* for each critical temperature and obtain more universal criteria for selecting DSS compositions, considering the peculiarities of alloying in each specific case, as well as the real heating rates, holding times, and cooling rates characteristic of specific industrial equipment.

## 4. Conclusions

It has been shown that for steels with similar PREN values, the corrosion resistance behavior under identical heat treatments can vary significantly. Depending on the amounts of carbonitride-forming elements, nitrogen, and niobium, the critical factor may be the phase balance between austenite and ferrite or the formation or dissolution of precipitates. This raises doubts about the applicability of the PREN for classifying DSSs and predicting their corrosion properties.

The redistribution of chemical elements between phases in DSSs was demonstrated, and the comparison with the calculation results indicated their high adequacy. Based on this, a method for calculating the *PREN*_*eff*_ is proposed, allowing for the consideration of the volume fractions of austenite and ferrite, taking into account the redistribution of nitrogen and carbon among precipitates and phases.

In practical examples, it was demonstrated that the behavior of critical points ($T_{50/50}^{\delta /\gamma }$ and $T_{{Cr}_{2}N}^{0}$) on the phase diagram fully describes the corrosion properties of DSSs. An approach for selecting DSS compositions was developed based on the positions of these critical points on the phase diagram. The maximum possible deviations from the ideal points $T_{50/50}^{\delta /\gamma }$ and $T_{{Cr}_{2}N}^{0}$ were defined, allowing for the selection of compositions and processing regimes, considering the incomplete progression of phase transformations in solid metals during actual production. 

This approach can be extended to other existing grades of DSSs and may serve as a reliable foundation for creating new grades.

## Figures and Tables

**Table 1:** Ranking of DSSs into groups based on the PREN values [12].

Group	PREN
Lean DSS	24 < PREN < 30
Standard DSS	30 < PREN < 40
Super DSS	40 < PREN < 48
Hyper DSS	PREN > 48

**Table 2:** The chemical compositions of the studied steels.

Steel	Element, wt.%											
	C	Si	Mn	Cr	Ni	Mo	Cu	N	Ti	Nb	V	PREN
1	0.018	0.4	0.4	26.7	6.7	4.2	0.5	0.13	0.01	0.01	0.02	43.4
2	0.015	0.6	1.6	24.3	6.1	4.1	3.0	0.20	0.01	0.35	–	46.8

## Data Availability

Data are contained within the article.
